# Effects of upper-body sprint-interval training on strength and endurance capacities in female cross-country skiers

**DOI:** 10.1371/journal.pone.0172706

**Published:** 2017-02-27

**Authors:** Kristine Vandbakk, Boye Welde, Andrea Hovstein Kruken, Julia Baumgart, Gertjan Ettema, Trine Karlsen, Øyvind Sandbakk

**Affiliations:** 1 Department of Sports Science and Physical Education, Nord University, Levanger, Norway; 2 School of Sport Sciences, UiT The Arctic University of Norway, Tromsø, Norway; 3 K.G. Jebsen Center of Exercise in Medicine, Department of Circulation and Medical Imaging, Faculty of Medicine and Health Science, NTNU, Norwegian University of Science and Technology, Trondheim, Norway; 4 Centre for Elite Sports Research, Department of Neuromedicine and Movement Science, Faculty of Medicine and Health Science, Norwegian University of Science and Technology, Trondheim, Norway; 5 St. Olav’s Hospital, Trondheim University Hospital, Trondheim, Norway; University of France Comté, FRANCE

## Abstract

This study compared the effects of adding upper-body sprint-intervals or continuous double poling endurance training to the normal training on maximal upper-body strength and endurance capacity in female cross-country skiers. In total, 17 female skiers (age: 18.1±0.8yr, body mass: 60±7 kg, maximal oxygen uptake (VO_2max_): 3.30±0.37 L^.^min^-1^) performed an 8-week training intervention. Here, either two weekly sessions of six to eight 30-s maximal upper-body double poling sprint-intervals (SIG, n = 8) or 45–75 min of continuous low-to-moderate intensity double poling on roller skis (CG, n = 9) were added to their training. Before and after the intervention, the participants were tested for physiological and kinematical responses during submaximal and maximal diagonal and double poling treadmill roller skiing. Additionally, we measured maximal upper-body strength (1RM) and average power at 40% 1RM in a poling-specific strength exercise. SIG improved absolute VO_2max_ in diagonal skiing more than CG (8% vs 2%, p<0.05), and showed a tendency towards higher body-mass normalized VO_2max_ (7% vs 2%, p = 0.07). Both groups had an overall improvement in double poling peak oxygen uptake (10% vs 6% for SIG and CG) (both p<0.01), but no group-difference was observed. SIG improved 1RM strength more than CG (18% vs 10%, p<0.05), while there was a tendency for difference in average power at 40% 1RM (20% vs 14%, p = 0.06). Oxygen cost and kinematics (cycle length and rate) in double poling and diagonal remained unchanged in both groups. In conclusion, our study demonstrates that adding upper-body sprint-interval training is more effective than continuous endurance training in improving upper-body maximal strength and VO_2max_.

## Introduction

Cross-country skiing involves upper-, lower- or whole-body exercise in the different techniques employed in varying terrain. It is one of the most demanding endurance sports, and elite cross-country skiers display some of the highest ever reported maximal oxygen uptake values (VO_2max_) [1–3]. Although VO_2max_ is most often reached in the diagonal (DIA) technique, peak oxygen uptake in other skiing techniques [3–5], as well as skiing efficiency [6–8] and upper-body power [9–12] are additional factors of importance for skiing performance.

In classical style cross-country skiing, DIA and double poling (DP) are the main techniques used in competitions, and for that reason they have been frequently used in the testing of elite skiers’ physiological capacities [13, 14]. Typically, skiers employ DP for skiing on flat terrain, while DIA is preferred on steeper uphills [15]. Since all propulsion in DP is exerted through the poles, the contribution of upper-body power to overall work in this technique is especially important. In fact, the upper-body produces approximately 50% of the external power in DP, and is additionally important in the transfer of potential and rotational energy (i.e., originally produced by the lower limbs in the repositioning phase) to poling power [16]. As sex differences in cross-country skiers become more pronounced with increasing power contribution from the upper-body [17], women may have a particularly large potential to improve their upper-body capacity and DP performance.

In a previous study, 20 weeks with added focus on continuous low-to-moderate intensity DP improved 10 km classical time-trial performance and gave large morphological and metabolic adaptations in the specific upper-body musculature in male elite cross-country skiers [18]. This highlights a large potential for adaptations in the upper-body of well-trained skiers. However, training at higher intensity could be more effective for boosting such endurance adaptations [19–21]. For example, in Zinner et al. [22] untrained men carried out six sprint-interval training (SIT) sessions with 4–6 x 30-s all-out sprints in an arm cranking and leg cycling mode. They concluded that the primary mechanism of adaptation to SIT in both modes was enhancement of aerobic energy production [22]. However, SIT was not compared to training at other intensities. While Nilsson et al. [23] revealed that six weeks of repeated 20-s maximal sprints in an upper-body mode improved work economy and 6-min performance to a similar extent as 180-s intervals, the only previous comparisons between SIT and continuous endurance training were done using leg exercise [24, 25]. These studies show that skeletal muscle and metabolic adaptations, as well as exercise performance, were improved to a similar extent in both training protocols. Hence, SIT may be a time-efficient strategy comparable to traditional endurance exercise with respect to training adaptations.

Therefore, the overall aim of this study was to compare the effects of adding upper-body sprint-intervals or continuous double poling endurance training to the normal training on maximal upper-body strength and endurance capacity in female cross-country skiers.

## Methods

### Participants

Initially, 21 highly-trained female junior cross-country skiers volunteered to participate in the study. Throughout the study period, four participants dropped-out or were excluded due to illness or insufficient compliance to training. Thus, in total 17 participants were included for the statistical analyses. The participants were students at two Norwegian high schools with a specialized program for cross-country skiing. All participants had regularly trained and competed in the sport of cross-country skiing for >5 years and ranged from being among the best juniors in the world to being top 30 in Norway. The participants mean age (18±1 yr), body-mass (60±7 kg), body height (166±5 cm) and training hours of the last season (504±92 hr) were not significantly different between groups at inclusion (all p<0.05).

### Ethics statement

The study was considered by the Regional Ethics Committee, Trondheim, Norway and approved by The Norwegian Data Protection Authority. All subjects signed an informed consent form before the experiment and were made aware that they could withdraw from the study at any point without providing an explanation. Five of the participants were below 18 years old and, consequently, one of their parents provided parental consent for participation in the study. The study was conducted in accordance with the Declaration of Helsinki.

### Procedures

The investigation took place from June 2014 to November 2014. In June, all participants were familiarized to test procedures and apparatus to minimize a learning effect from testing. Thereafter, skiers at one of the schools were allocated to a sprint-interval group (SIG, n = 8), whereas the skiers at the other school were designated to a control group (CG, n = 9). During an 8-week intervention period participants added either two weekly sessions of six to eight 30-s upper-body sprint intervals at maximal effort (SIG) or a 45–75 min session of continuous low-to-moderate intensity DP on roller skis (CG) to their normal training each week. Pre-testing was conducted in August and post-testing in October/ November using an identical 2-day protocol. On test day 1, maximal upper-body strength (1RM) and average power at 40% 1RM (P_40_) in a poling-specific strength exercise was measured. On test day 2, the participants were tested for physiological and kinematical responses during submaximal and maximal DIA and DP treadmill roller skiing. These two techniques are the most frequently employed classical techniques [13, 14] and they are used in different types of terrain [15]; DP on relatively flat terrain and DIA on steeper uphills. Another main reason for examining these two techniques is the difference in contribution from the upper-body, thereby allowing us to compare the effects of added upper-body training on modes where the arms and legs are of various importance. A standardized tapering period before the testing days was performed.

### Training intervention

In cooperation with the coaches at both schools, the pre-training (summer training) was standardized such that all skiers had approximately 1–2 high-intensity endurance sessions per week (these were normally executed as 2–8 min intervals or as simulated 15–30 min competitions at 88–97% of HR_max_), and 1 session per week with general strength exercises. The skiers did not perform SIT training or sessions with DP only during the summer. All skiers were instructed to carry out 2–3 sessions per week with roller-ski skating or classic skiing during the summer period. The total amount of training was individualized, meaning that the skiers did not all have exactly the same number of training hours. Low-intensity training accounted for the main individual differences in total training. During the intervention, the participants in SIG added two weekly sessions of six to eight 30-s upper-body sprint-intervals of maximum sustainable effort (isoeffort), separated by 2–3 min active rest (i.e. 15–20 min total work duration was logged as SIT for each session, see ref. 20). To facilitate variation in the training stimuli, the sprint-interval training was performed in two different modes each week; one while uphill DP on roller skis, and the other on a roller board in a kneeling position which has previously shown similar movement patterns and muscle activation as DP [12]. Each session included at least 20 min of a warm-up (~65% of maximal heart rate (HR_max_)). SIG progressively increased the number of intervals from six to eight throughout the intervention period. To facilitate sufficient resistance (high power output) in the roller board sessions throughout the period, the angle of the ramp was increased stepwise according to the athletes’ physical progression. The participants had to complete at least 12 (75%) out of 16 sprint-interval training-sessions to be included for the statistical analyses. The participants in the CG added 45–75 min sessions of continuous low-intensity DP on roller skis (60–81% of HR_max_). Training progression was accomplished by increasing the DP time from 45-min the first two training weeks, to 55-min in week 3–4, 65-min in week 5–6, and to 75-min the last two training weeks. These sessions were often incorporated in a 1.5–2 hours low-intensity classical roller skiing training. To be included for the statistical analyses, the participants had to complete at least 6 (75%) out of 8 DP sessions. Since we aimed to examine the actual effects of upper body sprint-intervals and not the effect of added upper-body load, we also added the load of the CG by increasing the amount of endurance training with an upper-body dominant mode (i.e, DP). We compared the SIT sessions with continuous low-to-moderate intensity DP by TRIMP-scores according to Bannister [26]. The calculated TRIMP-score for one session of 45–75 min of continuous DP was similar to two sessions of SIT with 6–8 repetitions of 30-sec sprints (i.e., TRIMP ranged between 3 and 4 for both sessions, dependent on the total time spent as DP and number of repetitions of SIT). This was in accordance with our pilot testing, where the athletes subjectively rated higher costs to perform the 45–75 min continuous DP sessions at low- to moderate-intensity than the 3–4 min of total work per SIT session. Overall, the TRIMP calculations and the subjects’ feedback provided the rational for adding 16 sprint-intervals and 8 continuous DP sessions. However, we are aware that the TRIMP score is not validated for upper-body work and we do not know if the heart rate used to classify upper-body DP is similar as compared to running or whole body exercise.

Training plans, a training diary, and written instructions about how to record training were provided and explained to the participants. Training data were recorded based on the participants own online training diaries (Olympiatoppens treningsdagbok, Lyymp AS, Norway). Intensity and type of exercise, including endurance, speed and strength training were registered as following: endurance training intensity was categorized into three intensity zones based on HR, according to a modification of the Norwegian Olympic system's intensity scale [27], with the methodology previously reported as valid [28]. Low-intensity training was performed at 60–81% of HR_max_, moderate-intensity training at 82–87% of HR_max_ and high-intensity training at 88–97% of HR_max_. Strength training was categorized into maximal strength training (≥85% 1RM) and submaximal strength training (<60% 1RM); speed training (<10 second) and SIT (30-s) were recorded including the breaks between sets.

### Test protocols

Each participant arrived in the laboratory at the same time of day for both trials. Over the 24 h preceding the first experimental trial, each participant had instructions of eating their normal diet as preparing to a sprint competition and the subjects replicated this diet before the second trial. Participants self-reported having not taken any supplements in the 1 month before or during the study. Subjects arrived for testing in a rested and hydrated state, at least 2 h postprandial and having avoided strenuous exercise, caffeine, and alcohol in the 24 h preceding the test sessions. Supplementation during the tests was restricted to sports drink.

#### Strength and power tests

The poling specific strength and power exercise test was performed while sitting on an upraised adjustable bench placed in front of a multi cable apparatus (Beach Mountain AS, Norway) with custom made grips and straps attached to the cord [29]. The back rest was at a ~120° angle with the seat. The skier sat on the bench with a ~90° angle at the knees and was strapped around the hips to isolate the upper-body. The friction in the pulldown apparatus, as measured with the Noraxon force cell, did not change with increasing weight. For the analysis of average power, a linear encoder was used (Muscle Lab Power, Ergotest Innovation AS, Porsgrunn, Norway) and the data processed with the associated computer software program (MuscleLab 3010E, software version 7.17; Ergotest Technology AS).

Warm-up consisted of 10-min running on a treadmill (~65% of HR_max_) followed by four sets of exercise-specific warm-up with gradually increasing load: two sets of ten repetitions at 40%, five repetitions at 60% and three repetitions at 80% of the expected 1RM [30]. The participants were holding a handlebar specifically designed to imitate the grip on poles in cross-country skiing, starting with the arms extended at shoulder level. During the last part of the pull-down the elbow joint should be extended more than 90° to be accepted, with the wrist reaching the trochanter major. The first attempt was performed with a load of 2.5 kg below the expected 1RM. After each successful attempt, the load was increased by 1 to 5 kg until the subject failed to lift the load correctly. The rest period between each attempt was 2 min. After a 10-min break, the average power (P_40_) in one pull-down at 40% of 1RM performed with maximal speed was measured. Two trials with two minutes of rest in between were performed and the best attempt was used for further analyses.

#### Treadmill tests

The roller ski tests were performed on a 2.5 x 3.5-m motor-driven treadmill (Rodby, Sodertalje, Sweden) while following security procedures described in detail previously [31]. To exclude possible variations in rolling resistance, all skiers used the same pair of Pro-ski classic roller skis with standard wheels (C2 Classic Pro-Ski, Sterners, Nyhammar, Sweden) and the same Rottefella binding system (Rottefella AS, Klokkartstua, Norway). For the determination of cycle characteristics during each test in each technique, a Sony video camera (Sony Handycam DCR-VX2000E, Sony Inc., Tokyo, Japan) was fixed on the side of the treadmill, enabling full view of the participants and the movement range of the poles. The video recordings were analyzed utilizing the Dartfish Pro 4.5 program (Dartfish Ltd, Fribourg, Switzerland). One cycle encompassed one right and one left pole push-off; cycle length was obtained by multiplying cycle time with the speed of the treadmill belt and cycle rate as the reciprocal of cycle time.

Respiratory variables were measured using open-circuit indirect calorimetry. Expired gas was passed through a mixing chamber and analyzed continuously (Oxycon Pro, Jaeger GmbH, Hoechberg, Germany). Before each test, the instruments were calibrated according to standard procedures. Heart rate (HR) was recorded continuously with a Polar S610i monitor (Polar Electro Oy, Kempele, Finland) synchronized with the Oxycon Pro system. Capillary blood lactate concentration (BLa) was measured using the validated Lactate Pro LT-1710*t* kit (ArkRay Inc, Kyoto, Japan) [32]. Rating of perceived exertion (RPE) was assessed utilizing the 6–20 point Borg Scale [33]. Body-mass was measured on the Kistler force plate (Kistler 9286AA, Kistler instrument Corp., Winterthur, Switzerland).

Following 10 min of treadmill warm-up (65% of HR_max_), each participant performed six 5-min stages of roller skiing at constant speed, alternating between the DP (at an incline of 3%) and DIA (12% incline) techniques (i.e., such that the six stages were performed in the order DP-DIA-DP-DIA-DP-DIA). A 2-min active break was given between each stage. The speed of the first stage corresponded to ~65% of participants’ maximal HR (6–8 km h^-1^ in DP and 5–6 km h^-1^ in DIA). The speed was increased by 2 km h^-1^ in DP and 1 km h^-1^ in DIA for each stage. The final minute at the speed of 10 km h^-1^ in DP and of 7 km h^-1^ in DIA was used for analyses of respiratory variables, in which oxygen cost determined work economy, as well as for examining cycle length and cycle rate according to calculations presented elsewhere [34]. Directly after completion of the test, BLa was assessed, and participants reported their RPE. This procedure was identical at pre- and post-testing.

The participants had 10 min of recovery after the submaximal tests before (1) completing an incremental test to exhaustion in DP, followed by a ~20 min recovery period (in which the last 3–5 min was easy DIA) and then (2) completed an incremental test to exhaustion in DIA. The recovery phase between the incremental tests was based on recovery time between final heats in cross-country sprint skiing competitions, and regarded long enough to prevent accumulation of fatigue [35, 36]. The incremental test in DP was performed with an initial speed of 12 km h^-1^ and increased by 2 km h^-1^ after the first minute up to 14 km h^-1^, and thereafter increased by 1 km h^-1^ every minute until exhaustion. During DIA, the initial speed of 8 km h^-1^ was increased by 1 km h^-1^ every minute until volitional exhaustion. Exhaustion was defined as the time-point at which the skiers were no longer able to keep the forefoot in front of a marker on the treadmill. Peak treadmill speed was calculated as v_f_ + [(*t*·*T*^-1^)·v_d_], where v_f_ was the velocity associated with the final workload, *t* the duration for which this maximal workload was maintained, *T* the duration of each individual level of workload, and v_d_ the difference in the velocities at which the final two workloads were performed [37]. Respiratory variables were continuously monitored and the average value of the three highest 10-s values measured successively defined as peak values. BLa was measured one minute after completion of each peak test and participants were immediately after asked to rate both muscular and cardiovascular exhaustion following the Borg RPE scale. Peak kinematics were measured during the last completed workload.

### Statistical analyses

All data were checked for normality by calculating *Z*-scores for skewness and kurtosis (criteria *Z*-value = -1.96<*Z*<1.96). Data are presented as mean and standard deviations (SD). Possible differences between groups at pre-test were checked by using an independent sample t-test procedure. Pre- to posttest changes within groups were tested by the paired samples t-test procedure. To investigate between group effects, univariate ANOVA with gain score (posttest-pretest) as the dependent variable, were performed. The between group differences at pre-test in one case, necessitated the use of an ANCOVA with pretest score as a covariate variable. For variables that did not meet the requirements for normality, within and between group effects were investigated by Wilcoxon Test and Mann-Whitney U Test procedures. Statistical significance was set at an alpha level of 0.05. All statistical analyses were performed as two-tailed tests. Repeated measurements of the physiological and kinematical variables used in this study showed intraclass correlation coefficients > 0.95. All statistical analyses were performed using the SPSS 21.0 Software for Windows (SPSS, Inc., Chicago, IL) and Office Excel 2010 (Microsoft Corporation, Redmond, WA).

## Results

### Training data

The average weekly distribution of training in the different exercise types is displayed in Table 1. There were no differences between SIG and CG in the time spent in different exercise modalities of endurance training, with 28 and 32% roller ski classic, 23 and 20% roller ski skating, 44 and 46% running and the remainder (5 and 3%) done in other modes such as cycling and kayaking, respectively. The compliance to the intervention was 83% in SIG and 77% in CG. The missing sessions were due to sickness (colds) and minor injuries and the compliance in both groups is regarded sufficiently high for such athletes; refraining from training when ill or injured reflects their normal life conditions as athletes. During the intervention, more SIT and speed training was conducted in SIG (both p<0.001), while CG performed more submaximal strength training and moderate-intensity training (both p<0.05). This was mainly due to one weekly session of DP added in the intervention. Rough retrospective training diary analyses of the 8-weeks prior to the beginning of the study assured that the basic training patterns did not differ from the intervention period, and that upper-body SIT and regular DP sessions were not performed in any of the groups preceding the intervention.

**Table 1:** Weekly endurance, strength, speed training, plyometric and mobility training during the 8-week intervention training period in 17 highly trained female cross country skiers (hh:mm and percent).

	Sprint interval group (SIG, n = 8)		Control group (CG, n = 9)	
	Mean±SD	%	Mean±SD	%
Low-intensity training	9:48±1:57	72.9	10:10±2:54	73.1
Moderate-intensity training	0:25±0:08	3.1	0:37±0:14^#^	4.4
High-intensity training	0:38±0:07	4.6	0:36±0:10	4.3
Sprint-interval training	0:30±0:11^##^	3.7	0:00±0:01	0.0
Maximal strength training	0:32±0:09	4.0	0:45±0:13	5.4
Submaximal strength training	0:43±0:24	5.3	1:08±0:19^#^	8.2
Speed training	0:22±0:04^##^	2.7	0:08 ± 0:07	1.0
Plyometrics	0:09±0:22	1.1	0:14±0:13	1.7
Mobility training	0:13±0:21	1.6	0:08±0:07	1.0
Other	0:07±0:06	0.9	0:08 ± 0:15	1.0
TOTAL	13:27±2:26	100.0	13:54±3:44	100.0

SIG, sprint-interval training group; CG, control group; Low-intensity training at 60–81% of HR_max_; Moderate-intensity training at 82–87% of HR_max_; High-intensity training at 88–97% of HR_max_; Sprint-interval training with all-effort bouts of approx. 30-s; Maximal strength training at ≥85% of 1RM (4–5 repetition, 3–5 set with >2min rest between); Submaximal strength training at <60% of 1RM (12–18 repetitions, 3–4 set with 1–1.5 min rest between); Speed training with all-effort bouts of <10-s. Significant differences between training groups during the intervention period

^#^p<0.05

^##^p<0.001.

### Strength and power tests

**Fig 1:**
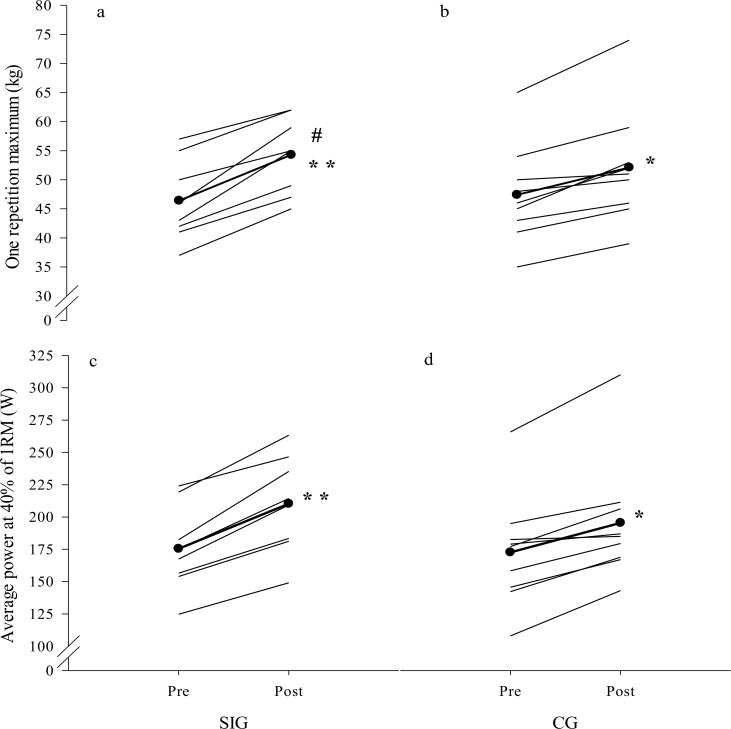
**Individual data points for one repetition maximum before (a) and after the intervention period (b) and for average power at 40% of one repetition maximum before (c) and after the intervention period (d) for the upper-body sprint-interval training group (SIG) and the control group (CG).** Mean values are represented by the thick line with black circles. Significant within-group changes: *p<0.05, **p<0.001; Significant different change from pre to post between groups: ^#^p<0.05.

There were no significant changes in body-mass from pre to post-test neither within nor between groups (CG: +0.3±1.5 kg, SIG: +0.9±1.1 kg) (all p>0.05). Pre- and post-test scores for 1RM and P_40_ are shown in **Fig 1A–1D**. The 1RM improved more for SIG than CG with significant increases within both groups (18% (7.9±3.0 kg) versus 10% (4.7±2.7 kg), all p<0.035). P_40_ tended to increase more in SIG than CG (20% (34.9±11.1 W) versus 14% (22.8±13.0 W), p = 0.057) with significant increases within both groups (both p<0.001).

### Roller ski tests

**Fig 2:**
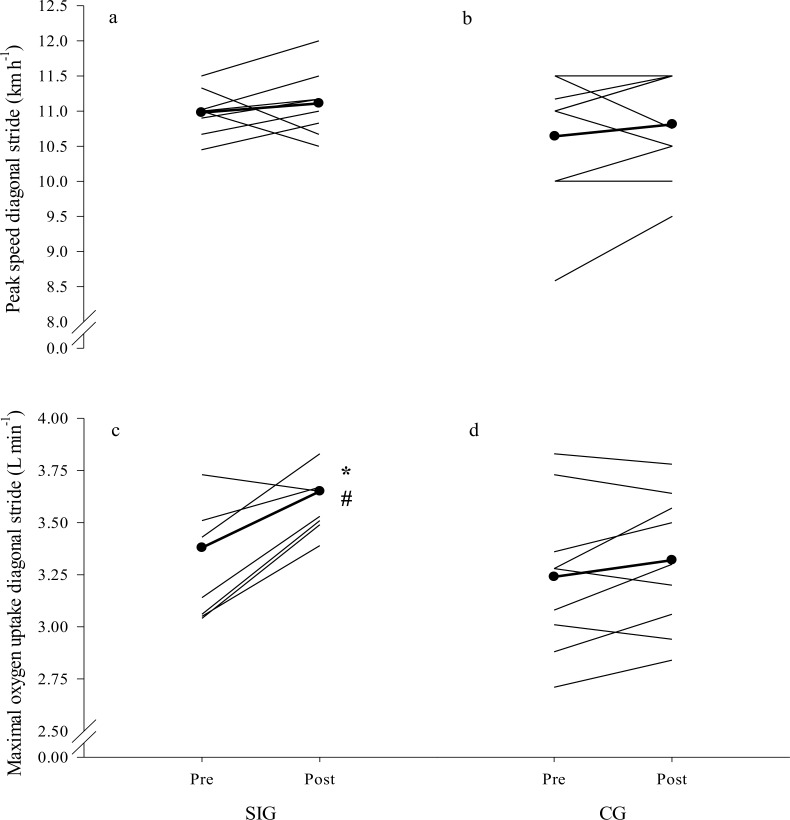
**Individual data points for peak velocity in diagonal stride before (a) and after the intervention period (b) and for peak oxygen uptake in double poling before (c) and after the intervention period (d) for the upper-body sprint-interval training group (SIG) and the control group (CG).** Mean values are represented by the thick line with black circles. Significant within-group changes: *p<0.05.

**Fig 3:**
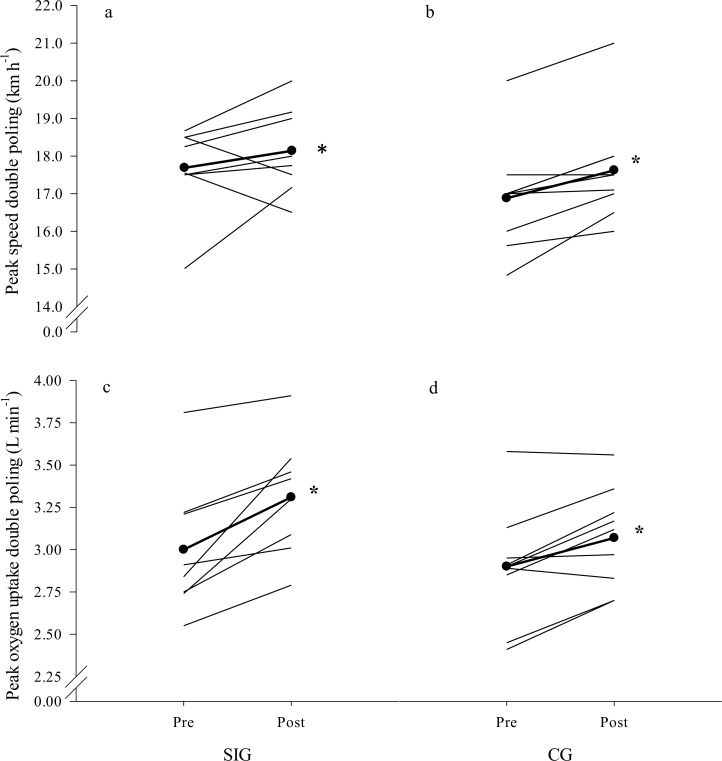
**Individual data points for peak velocity in double poling before (a) and after the intervention period (b) and for maximal oxygen uptake in diagonal stride before (c) and after the intervention period (d) for the upper-body sprint-interval training group (SIG) and the control group (CG).** Mean values are represented by the thick line with black circles. Significant within-group differences: *p<0.05; Significant different change from pre to post between groups: ^#^p<0.05.

Maximal physiological and kinematical responses at pre-and post-tests conditions are displayed for DIA in **Table 2** and **Fig 2A–2D** and for DP in **Table 3** and **Fig 3A–3D**. SIG exhibited a greater increase (p<0.05) in absolute values and a trend (p<0.07) towards larger increase in body-mass normalized VO_2max_ in DIA when compared to CG (9% and 7% increase in SIG versus 2% and 2% in CG). No significant between-group differences for absolute or body-mass normalized VO_2peak_ in DP, or for any of the other physiological variables in DIA or the DP were found. Within the groups, peak ventilation in DIA increased by 6% from pre- to post-test in SIG, whereas in DP, 2 and 4% higher peak treadmill speeds and 10 and 6% higher VO_2peak_ were revealed in SIG and CG, respectively, and peak HR and peak BLa increased significantly from pre- to post-test in CG (all p<0.05). The CG increased their time to exhaustion on the maximal test in DP by 18% from pre- to post-test (p = 0.002). No significant within- or between-groups changes were observed from pre- to post-test in time to exhaustion on the maximal test in DIA.

**Table 2:** Maximal physiological and kinematical responses at pre-and post-tests conditions in diagonal stride (DIA) treadmill roller skiing (12% incline) for 17 highly trained female junior cross-country skiers.

	SIG (n = 8)		CG (n = 9)		SIG vs. CG	
Variables	Pre	Post	Pre	Post	Betwen-group effects	
	Mean±SD	Mean±SD	Mean±SD	Mean±SD	F-value	Sig.
Time to exhaustion (s)	240±21	246±29	218±57	228±47	F_(1,15)_ = 0.04	P = 0.85
VO_2_ (ml∙kg^-1^∙min^-1^)	56.1±2.9	59.9±3.9*	53.8±4.7	54.8±4.4	F_(1,15)_ = 4.0	P = 0.07
VE (L∙min^-1^)	127.8±15.2	135.8±12.7*	113.2±14.1	117.4±9.8	F_(1,15)_ = 0.9	P = 0.40
RER	1.16±0.04	1.14±0.08	1.11±0.06	1.09±0.04	F_(1,15)_<0.01	P = 0.95
HR (bpm)	190.6±5.9	190.1±3.2	190.3±3.8	189.8±3.1	F_(1,15)_<0.01	P = 0.98
RPE (6–20)	19.6±0.7	19.4±0.7	19.3±0.5	19.2±0.8	F_(1,15)_ = 0.1	P = 0.74
Bla (mmol∙L^-1^)	10.88±1.97	10.78±1.64	9.41±2.26	10.31±2.94	F_(1,15)_ = 1.5	P = 0.24
CR (Hz)	0.92±0.08	0.91±0.04	0.92±0.05	0.93±0.05	F_(1,14)_ = 0.7	P = 0.43
CL (m)	3.35±0.28	3.51±0.16	3.25±0.28	3.32±0.18	F_(1,14)_ = 0.7	P = 0.41

SIG, sprint-interval training group; CON, control group; VO_2_, oxygen uptake; VE, ventilation; RER, respiratory exchange ratio; HR, heart rate; RPE, rating of perceived exertion; BLa, blood lactate concentration; CR, cycle rate; CL, cycle length. Within-group differences

*p<0.05.

**Table 3:** Maximal physiological and kinematical responses at pre-and post-tests conditions during double poling (DP) treadmill roller skiing (3% incline) for 17 highly trained junior female cross-country skiers.

	SIG (n = 8)		CG (n = 9)		SIG vs. CG	
Variables	Pre	Post	Pre	Post	Betwen-group effects	
	Mean±SD	Mean±SD	Mean±SD	Mean±SD	F-value	Sig.
Time to exhaustion (s)	341±69	368±70	286±95	337±86**	F_(1,15)_ = 1.0	P = 0.33
VO_2_ (ml∙kg^-1^∙min^-1^)	49.8±2.8	54.3±4.5*	48.1±4.9	50.8±4.1*	F_(1,15)_ = 1.5	P = 0.25
VE (L∙min^-1^)	118.0±16.4	128.0±15.2	108.8±15.5	111.0±11.4	F_(1,15)_ = 1.6	P = 0.23
RER	1.11±0.05	1.09±0.08	1.09±0.03	1.07±0.04	F_(1,15)_ = 0.1	P = 0.78
HR (bpm)	189.4±4.5	191.5±4.0	189.4±4.4	192.3±2.8*	F_(1,15)_ = 0.2	P = 0.67
RPE (6–20)	18.5±1.2	18.8±1.3	18.7±0.9	18.6±0.7	F_(1,15)_ = 0.5	P = 0.51
Bla (mmol∙L^-1^)	8.70±2.00	9.53±1.03	8.34±1.74	9.88±2.33*	F_(1,15)_ = 0.6	P = 0.46
CR (Hz)	1.05±0.15	1.11±0.12	1.16±0.11	1.19±0.15	F_(1,14)_ = 0.3	P = 0.59
CL (m)	4.82±0.60	4.61±0.65	4.05±0.48	4.19±0.81	F_(1,14)_ = 2.4	P = 0.15

SIG, sprint-interval training group; CG, the control group; VO_2_, oxygen uptake; VE, ventilation; RER, respiratory exchange ratio; HR, heart rate; RPE, rating of perceived exertion; BLa, blood lactate concentration; CR, cycle rate; CL, cycle length. Within-group differences

*p<0.05

**p<0.01.

Submaximal physiological and kinematical responses at pre- and post-tests conditions are displayed for DIA in **Table 4** and for DP in **Table 5.** In DP, the changes from pre- to post-test significantly differed between groups in absolute VO_2_, VE and RER (all p<0.05). This coincided with 6–7% increases in absolute VO_2_ and VE in SIG and a decrease of 0.05 in RER in CG (all p<0.05). No other between-or within-groups differences were revealed.

**Table 4:** Submaximal physiological and kinematical responses at pre- and post-tests conditions in diagonal stride (DIA) treadmill roller skiing (12% incline) at standardized speed (7 km∙h^-1^) for 17 highly trained junior female cross-country skiers.

	SIG (n = 8)		CG (n = 9)		SIG vs. CG	
Variables	Pre	Post	Pre	Post	Betwen-group effects	
	Mean±SD	Mean±SD	Mean±SD	Mean±SD	F-value	Sig.
VO_2_ (ml∙kg^-1^∙min^-1^)	44.6±2.2	44.4±2.1	42.2±1.5	42.3±1.3	^b^F_(1,15)_ = 2.7	P = 0.13
VO_2_ (L∙min^-1^)	2.69±0.27	2.71±0.19	2.55±0.32	2.57±0.30	F_(1,15)_ = 0.01	P = 0.92
VE (L∙min^-1^)	77.9±15.0	73.3±10.5	69.4±11.1	67.4±8.8	F_(1,15)_ = 0.5	P = 0.49
RER	0.93±0.04	0.92±0.02	0.93±0.04	0.88±0.04*	F_(1,15)_ = 2.1	P = 0.17
HR (bpm)	165.4±16.3	172.6±7.9	174.3±9.1	175.8±5.1	F_(1,15)_ = 1.0	P = 0.34
RPE (6–20)	13.8±3.3	12.5±2.6	14.2±2.1	13.6±2.1	F_(1,15)_ = 0.4	P = 0.53
Bla (mmol∙L^-1^)	3.35±2.11	2.89±1.18	3.39±2.10	2.36±0.84	^a^Z = -0.58	P = 0.61
CR (Hz)	0.70±0.06	0.68±0.06	0.77±0.04	0.76±0.07	^a^Z = -0.10	P = 0.96
CL (m)	2.79±0.26	2.87±0.25	2.54±0.13	2.56±0.24	^a^Z<0.01	P = 1.00

SIG, sprint-interval training group; CG, control group; VO_2_, oxygen uptake; VE, ventilation; RER, respiratory exchange ratio; HR, heart rate; RPE, rating of perceived exertion; BLa, blood lactate concentration; CR, cycle rate; CL, cycle length. Within-group differences

*p<0.05.

^a^Based on Mann-Whitney U test (see the statistical section for details).

^b^: Based on ANCOVA, with pretest score as a covariate variable, due to between group differences in pre-test. NA: Not available due to use of Mann-Whitney U test (see the statistical section for details).

**Table 5:** Submaximal physiological and kinematical responses at pre- and post-tests conditions during double poling (DP) treadmill roller skiing (3% incline) at standardized speed (10 km∙h^-1^) for 17 highly trained junior female cross-country skiers.

	SIG (n = 8)		CG (n = 9)		SIG vs. CG	
Variables	Pre	Post	Pre	Post	Betwen-group effects	
	Mean±SD	Mean±SD	Mean±SD	Mean±SD	F-value	Sig.
VO_2_ (ml∙kg^-1^∙min^-1^)	29.3±2.3	30.7±2.2	30.5±3.0	30.0±2.2	F_(1,15)_ = 3.6	P = 0.08
VO_2_ (L∙min^-1^)	1.77±0.27	1.88±0.22*	1.84±0.24	1.82±0.20	F_(1,15)_ = 5.2	P = 0.04
VE (L∙min^-1^)	52.8±7.3	57.1±8.6*	56.0±7.3	53.0±7.0	F_(1,15)_ = 10.0	P = 0.01
RER	0.97±0.07	0.97±0.05	0.98±0.04	0.93±0.05*	F_(1,15)_ = 5.6	P = 0.03
HR (bpm)	165.4±16.3	172.6±7.9	174.3±9.1	175.8±5.1	F_(1,15)_<0.01	P = 0.97
RPE (6–20)	11.0±2.9	10.5±3.2	12.3±2.0	12.0±2.1	^a^Z = -0.75	P = 0.48
Bla (mmol∙L^-1^)	2.69±1.02	2.98±1.47	2.92±1.00	2.79±0.74	F_(1,15)_ = 1.2	P = 0.30
CR (Hz)	0.70±0.12	0.71±0.12	0.74±0.07	0.73±0.08	^a^Z = -0.53	P = 0.60
CL (m)	4.07±0.66	4.00±0.64	3.81±0.38	3.82±0.45	^a^Z = -0.39	P = 0.74

SIG, sprint-interval training group; CG, control group; VO_2_, oxygen uptake; VE, ventilation; RER, respiratory exchange ratio; HR, heart rate; RPE, rating of perceived exertion; BLa, blood lactate concentration; CR, cycle rate; CL, cycle length. Within-group differences

*p<0.05.

^a^: Based on Mann-Whitney U test (see the statistical section for details). NA: Not available due to use of Mann-Whitney U test (see the statistical section for details).

## Discussion

This study compared the effects of adding upper-body sprint-intervals or continuous double poling endurance training to the normal training on maximal upper-body strength and endurance roller ski capacity among female cross-country skiers. The main findings were that increased emphasis on sprint-intervals showed greater improvements in VO_2max_ in DIA and upper-body maximal strength than continuous endurance training. However, significant improvements in DP peak speed and VO_2peak_, as well as in upper-body strength and power were found within both groups, although large individual variations in the adaptations were present.

### Training during the study

The compliance of the scheduled training in the intervention period reached an acceptable level for inclusion in statistical analyses in both groups. In the intervention period, the overall training patterns mainly differed as planned throughout the intervention; SIG added the sprint-intervals, whereas CG trained more low- to moderate-intensity training as DP. However, we retrospectively found two additional differences between the groups; more speed training was performed in SIG, which could have supplemented the effects of the upper-body sprint-intervals on developing neuromuscular characteristics and strength/power characteristics. In addition, more submaximal strength training was performed by CG. This may be a limitation of our design. However, our retrospective training diary analyses assured that the only difference from the training performed 8 weeks prior to the intervention within SIG was the experimental training during the intervention. Correspondingly in CG, moderate-intensity training was the only training that increased in the intervention period. Although these findings strongly indicate that the differences in training between groups are valid for the current research questions, our study also highlights the difficulties of manipulating only one variable in a real-life context in elite athletes.

Our aim was to investigate the added effects of upper body sprint-intervals to traditional approaches of improving upper-body endurance capacity, and not the effect of added upper-body load *per se*. Thus, we included one weekly session of continuous low- to moderate-intensity DP in the CG. In the absence of a valid model to match the different training sessions for total training load, we pilot tested the different sessions on athletes with comparable levels as our test subjects. Much more time was spent on each added training sessions for CG (45–75 min of work vs. 3–4 min of total work per session within 15–20 min in SIG) and the athletes felt that the continuous sessions at low- to moderate-intensity double poling were more demanding than the sprint-intervals. Therefore, we added 16 sprint-intervals and 8 continuous sessions. This added load was regarded feasible in both groups, and is comparable to previous investigations where aerobic high-intensity interval training has been compared to moderate continuous exercise [24, 25].

### Upper-body strength and power

A main finding of this study was the larger increase in maximal upper-body strength in SIG compared to CG, and the coinciding trend for P_40_. SIG improved strength and power by ≈18–20% which was in accordance with the 15% strength gains observed following a 9-week maximal upper-body strength regime among female cross-country skiers [38]. Hence, the 30-s upper-body sprint-interval sessions with emphasis on maximal mobilization seem highly effective for both strength and power gains in this population of cross-country skiers.

However, also CG revealed considerable strength gains as a result of greater emphasis on upper-body endurance training in the DP mode. This may be explained by improved coordination of neuromuscular capacities within the DP movement due to more training in this technique [39]. Overall, the responses found here may be explained by underdeveloped upper-body muscles of our female junior cross-country skiers, which thereby provided a large potential for further improvements independent of added stimuli.

### Performance and peak aerobic capacity

Another main finding of this study was the 6% larger increase in DIA VO_2max_ for SIG compared to CG, indicating that upper-body sprint-intervals may be superior to continuous endurance exercise for improving VO_2max_. However, this finding approached significance between groups only for absolute values of VO_2max_, which can probably be explained by the non-significant increase in body mass in SIG (i.e., ≈ 0.6 kg more than for CG from pre- to post-test). This is supported by a previous study, where Zinner et al. [22] showed a significant increase in VO_2peak_ after SIT training. However, our data contrasts a study done on well-trained individuals employing leg exercise where unchanged VO_2max_ values followed sprint-interval training [40]. Nevertheless, improvements in VO_2max_ by adding upper-body sprint-intervals are supported by studies on recreational active participants, during two-leg cycling [24, 41–43]. While our participants were highly trained in their lower body, the large potential for further improvements of the upper body of skiers is previously shown in a study by Terzis et al. [18], where well-trained male cross-country skiers extensively increased their upper body training through greater emphasis on double poling during roller-skiing; the skiers who demonstrated the largest improvement in performance also exhibited the largest muscle adaptations. This potential may be particularly large in women as previous studies reveal that the performance gap compared to men is enhanced when the contribution from the upper-body increases [44, 45]. Taken together, the junior skiers in our study showed a large potential to improve their upper-body capacity. Sprint-interval training may, therefore, have enabled them to use their whole-body more effectively during DIA and thereby reach higher VO_2max_ values. However, the fact that changes in roller ski performances or DP VO_2peak_ did not differ between groups calls for an alternative explanation; for example that continuous endurance training may induce more peripheral adaptations in the upper-body musculature, while the higher intensity of sprint-intervals may induce more central adaptations associated with VO_2max_ increases [46].

The magnitude of improvements in DP roller ski performance (i.e., peak treadmill speed) and VO_2peak_ were similar as seen in DIA, and our two groups showed similar improvements on these parameters in DP. The only comparable study using upper-body sprint-intervals is done by Nilsson et al. [15] who showed positive effects on power output in a 6-min test on a DP ergometer after 6 weeks of training. However, in that study the effects of sprint-interval training did not differ from another group using longer intervals. Furthermore, Terzis et al. [18] showed that long duration, low-to-moderate intensity workouts using DP resulted in improved performance in a 10 km classical time-trial and induced large morphological and metabolic adaptations of the triceps brachii muscle. Altogether, these studies indicate that more upper-body training in general may be favorable in order to improve performance and aerobic capacity in DP.

### Submaximal oxygen cost and kinematics

Nilsson et al. [23] found a positive effect of upper-body sprint-intervals on work economy in a DP ergometer. However, in our study SIG actually increased the absolute oxygen cost of DP whereas the body mass normalized oxygen cost of submaximal roller skiing remained unchanged from pre-to post-testing for DP and DIA in both groups. The increased oxygen cost of DP was partly attributed to small but non-significant changes in body-mass from pre to post in SIG. However, a tendency towards worse DP economy was also present for the body mass normalized values. The reason for this contradiction with the previous study [15] is unknown and requires further elucidation.

We found unchanged kinematical responses for both groups and techniques, in which a reasonable explanation would be that a modification of established cycle characteristics does not occur by an 8-week training period. However, in DP we would have expected systematic improvements in kinematic patterns at peak speed since this was increased from pre- to post-test. The absence of this is explained by different strategies used by the skiers; some increased DP speed by increasing cycle rate and others by increased cycle length.

### Limitations of the study

We standardized the training preceding the study and emphasized the initial physical fitness evaluation and laboratory familiarization. These procedures were employed to minimize learning effects from pre- to post-test. In addition, the initial evaluation of each subject was essential to assure two equal training groups since no randomization procedure was used. This latter aspect might be regarded a weakness of our design; however, one training regime at each of the schools was chosen to ensure that the same basic training pattern was followed during the entire intervention period.

## Conclusions

This present study demonstrates that adding upper-body sprint-interval training is more effective than adding continuous endurance training for improving upper-body maximal strength and VO_2max_ in DIA. However, the considerable improvements in DP peak speed, VO_2peak_ and upper-body strength and power found within both groups indicate that female junior cross-country skiers have a large potential to further improve their upper-body capacities independent of the nature of the stimuli. Hence, depending on the aim of training, both upper-body sprint-intervals and endurance training may successfully be added to female cross-country skiers’ training program.

## Supporting information

S1 File Supporting data. (XLSX)
